# Age-related resistance of skeletal muscle-derived progenitor cells to SPARC may explain a shift from myogenesis to adipogenesis

**DOI:** 10.18632/aging.100426

**Published:** 2012-01-30

**Authors:** Serena Katsuyuki Nakamura, Shin-ichi Nakano, Takahiro Miyoshi, Keitaro Yamanouchi, Takashi Matsuwaki, Masugi Nishihara

**Affiliations:** Department of Veterinary Physiology, Graduate School of Agricultural and Life Sciences, The University of Tokyo, 1-1-1 Yayoi, Bunkyo-ku, Tokyo 113-8657, Japan

**Keywords:** aging, SPARC, skeletal muscle, adipogenesis, myogenesis, differentiation, integrin α5

## Abstract

Aging causes phenotypic changes in skeletal muscle progenitor cells (SMPCs) that lead to the loss of myogenicity and adipogenesis. Secreted protein acidic and rich in cysteine (SPARC), which is secreted from SMPCs, stimulates myogenesis and inhibits adipogenesis. The present study aimed to examine whether changes in SPARC expression, its signaling pathway, or both are involved in age-related phenotypic changes in SMPCs. SPARC expression levels were comparable in SMPCs derived from young and old rats. However, when SPARC expression was reduced by a SPARC-specific siRNA, SMPCs from young rats showed reduced myogenesis and increased adipogenesis. In striking contrast, old rats showed little changes in these functions. Recombinant SPARC was effective in inhibiting adipogenesis and promoting myogenesis of SMPCs from young rats but had no effect on SMPCs from old rats when endogenous SPARC levels were reduced by the SPARC-siRNA. Further, the level of integrin α5, a subunit of the putative SPARC receptor, was decreased in SMPCs from old rats, and its inhibition in SMPCs from young rats by siRNA reduced adipogenesis in response to SPARC. These results suggest that, although SPARC plays a role in regulating SMPC function, SMPCs become refractory to the action of SPARC with age. Our data may explain an age-related shift from myogenesis to adipogenesis, associated with sarcopenia.

## INTRODUCTION

Aging impairs organ function. Human body mass, of which skeletal muscle is the most abundant tissue, declines with age [1]. The decline in skeletal muscle (sarcopenia) is defined clinically by a loss of lean muscle mass and impairment of its function [2]. Adipose tissue infiltration is often observed in skeletal muscles of the elderly [3], and this intramuscular adipose tissue (IMAT) causes poor physical performance [4] and plays a role in insulin resistance and obesity [5]. Therefore, treating these conditions as well as sarcopenia will depend on a better understanding of the mechanism of IMAT accumulation.

Skeletal muscle contains several types of SMPCs, including satellite cells that possess almost exclusively myogenic potential [6]. Adult mouse SMPCs spontaneously differentiate into adipocytes [7] and their adipogenic potential increases with age [8]. Vettor, et al. postulate that SMPCs form IMAT [9], thus suggesting that alterations in the differentiation of SMPCs cause sarcopenia. Differentiation of SMPCs is greatly affected by their microenvironment [10, 11]. For example, degradation of basal lamina, a major component of the SMPC niche, promotes adipogenic differentiation of SMPCs [11]. Basal lamina contains several extracellular matrix (ECM) proteins such as collagen, laminin, fibronectin and proteoglycans. ECM proteins function as structural supports for SMPCs and bind growth factors that regulate SMPC function [12].

SPARC (also known as osteonectin or BM-40) is a nonstructural, matricellular glycoprotein that is secreted into the niche and functions in cell adhesion, angiogenesis, growth factor binding, and cell differentiation [13, 14]. SPARC inhibits adipogenesis of preadipocytes by enhancing β-catenin signaling [15]. It also promotes myogenic differentiation of C2C12 and MM14 myoblasts [16, 17]. SPARC is expressed by SMPCs [18] and may regulate SMPC function in an autocrine manner. SPARC-null mice exhibit early-onset cataracts [19], osteopenia-like decline of bone formation [20], increased obesity, and decreased skeletal muscle mass [21]. These phenotypes are typically observed in old mice.

In the present study, we examined whether changes in SPARC expression or signaling are involved in age-related phenotypic changes during the differentiation of SMPCs.

## RESULTS

### SMPCs express SPARC regardless of their differentiation state during myogenesis and adipogenesis

**Figure 1:**
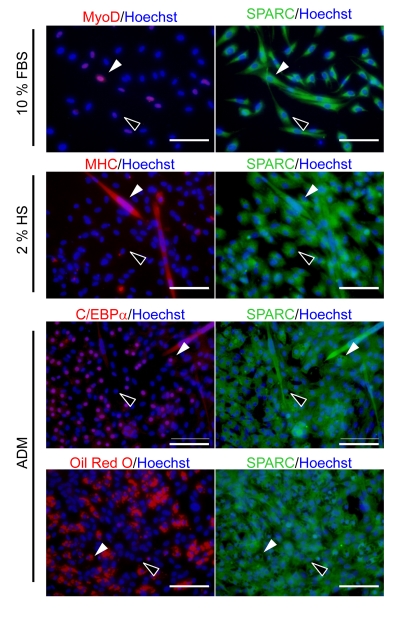
Immunocytochemistry of SPARC in SMPCs Immunostaining of SPARC during myogenic and adipogenic differentiation of SMPCs from young rats. Cells were co-stained with anti-MyoD and anti-SPARC 3 days after plating or with anti-MHC and anti-SPARC 4 days after exposure to media containing 2% HS. Co-staining with anti-SPARC and anti-C/EBPα or Oil Red O staining was performed 4 days after culturing in the media to induce adipogenic differentiation (ADM). Nuclei were visualized with Hoechst 33258 Dye (blue). White arrowheads indicate double-positive cells (MyoD+/SPARC+, MHC+/SPARC+, C/EBPα+/SPARC+, and Oil Red O+/SPARC+), whereas black arrowheads indicate cells that are only positive for SPARC (MyoD-/SPARC+, MHC-/SPARC+, C/EBPα-/SPARC+, and Oil Red O-/SPARC+). Scale bar = 100 μm.

SMPCs express SPARC [18] and can differentiate into myogenic and adipogenic cells [7]. To examine whether the expression of SPARC by SMPCs is differentiation dependent, we immunostained SMPCs derived from young rats by using antibodies specific for MyoD, C/EBPα, myosin heavy chain (MHC), and SPARC (Figure 1). MyoD is a transcription factor that plays a critical role in myogenesis. MyoD-positive cells coexpressed SPARC in SMPC cultures 3 days after plating. MHC-positive myotubes, induced by culturing SMPCs in media containing 2% HS, were also positive for SPARC. Approximately 20% of SMPCs expressed C/EBPα-, a transcription factor involved in adipogenesis, and were stained with Oil Red-O, an indicator of adipogenesis. Both C/EBPα- and Oil Red-O-positive cells expressed SPARC. In all cases, SPARC expression was detected in both non-myogenic and non-adipogenic cells, indicating that SMPC express SPARC regardless of their state of differentiation.

### SPARC expression levels in SMPCs do not change with age

**Figure 2:**
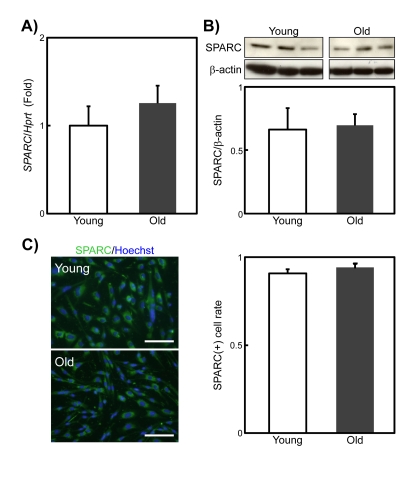
SPARC expression in young and old rat SMPCs (**A**) qPCR analysis of SPARC expression in SMPCs from young and old rats 3 days after plating. Data are expressed as the mean ± standard error of the mean (SEM) (n = 4, respectively). (**B**) Immunoblotting analysis of SPARC expression levels in SMPCs from young and old rats 3 days after plating. Data are expressed as mean ± SEM (n = 3, respectively). (**C**) Representative immunostaining experiments to detect SPARC expression in SMPCs from young and old rats 3 days after plating (left). Scale bar = 100 μm. A plot of the proportions of nuclei in SPARC-positive cells is shown (right). Data are expressed as the mean ± SEM (n = 3, respectively).

The adipogenic potential of SMPC increases with age [8], which we confirmed here (data not shown). Because SPARC inhibits adipogenesis [15], the age-related increases in the adipogenic potential of SMPCs may be due to a decline in their secretion of SPARC. However, quantitative PCR analysis revealed no differences in SPARC expression levels between SMPCs from young and old rats (Figure 2A). Immunoblotting and immunostaining of SPARC in young and old SMPCs also showed that the levels of SPARC expression in SMPCs does not change with age (Figures 2B and 2C). These results suggest that the increased adipogenic potential of old SMPCs is not caused by a decline in SPARC.

### The efficiency of RNA silencing of SPARC is independent of SMPC age

**Figure 3:**
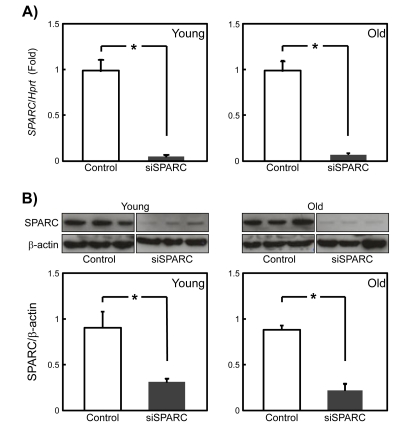
Inhibition of SPARC expression by RNA silen-cing in young and old rat SMPCs (**A**) The efficiency of silencing by siSPARC was evaluated by qPCR in SMPCs from young and old rats 2 days after siRNA transfection. Data are expressed as the mean ± SEM (n = 4, respectively). *p < 0.05 vs. control. (**B**) The efficiency of siSPARC silencing was evaluated by immunoblotting SMPC extracts prepared from young and old rats 2 days after siRNA transfection. (**C**) Quantitative analysis of immunoblotting data. Data are expressed as mean ± SEM (n = 3, respectively). *p < 0.05 vs. control.

Despite the anti-adipogenic function of SPARC [15], there was no difference in the expression of SPARC as a function of SMPC age. This raises the possibility that responsiveness of SMPCs to SPARC, rather than its expression, declines with age. To examine this, we employed the siRNA for SPARC (siSPARC) in an attempt to down-regulate SPARC. First, we determined the knockdown efficiency of siSPARC in young and old SMPCS by qPCR and immunoblotting. One day after siSPARC transfection, SPARC gene expression was silenced by more than 90% in young and old SMPCs (Figure 3A). Immunoblotting revealed that the amount of SPARC was reduced to a similar extent in cells derived from young and old rats (Figure 3B). These results indicate that the efficiency of siRNA-mediated knockdown of SPARC did not change in SMPCs with age.

### Effects of SPARC knockdown on the differentiation of SMPCs diminish with age

**Figure 4:**
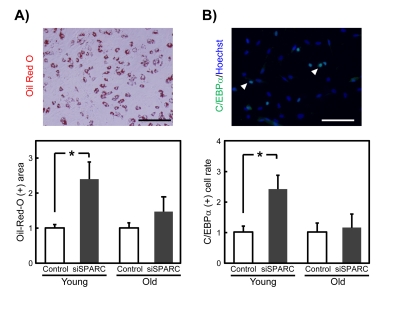
Effects of siSPARC on the adipogenic potential of SMPCs (**A**) Representative Oil Red-O staining of SMPCs cultured for 4 days in differentiation medium (above), and quantitative analysis of Oil Red-O-positive area (below). Oil Red-O-positive areas were quantified as pixel values and the ratios to control values are graphed. Data are expressed as mean ± SEM (n = 4, respectively). *p < 0.05 vs. control. Scale bar = 200 μm. (**B**) Representative immunostaining of C/EBPα in SMPCs cultured for 4 days in differentiation media (above), and quantitative analysis of C/EBPα-positive cells (below). Nuclei were counterstained with Hoechst 33258. White arrowheads indicate C/EBPα-positive nuclei. Scale bar = 100 μm. The proportions of C/EBPα-positive cells were calculated and the ratios to control values are graphed. Data are expressed as mean ± SEM (n = 4, respectively). *p < 0.05 vs. control.

We next evaluated the adipogenic potential of young and old SMPCs when SPARC expression was reduced by its cognate siRNA (Figure 4). In young SMPCs transfected with siSPARC, the Oil Red-O-positive area was increased, suggesting that SPARC suppresses adipogenesis (Figure 4A). In contrast, no changes were observed in old SMPCs. Adipogenesis was also evaluated by immunostaining for C/EBPα, a transcription factor whose levels increase with adipogenesis. The results were similar to those for Oil Red-O staining (Figure 4B), suggesting that endogenously produced SPARC inhibits adipogenesis of SMPC, but this effect diminishes with age, presumably due to the loss of SPARC function.

**Figure 5:**
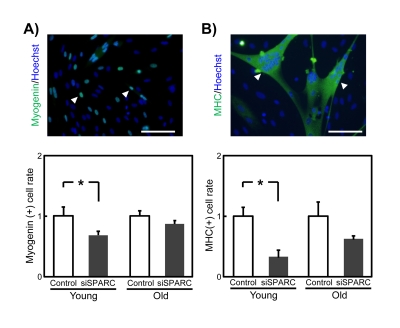
Effects of siSPARC on the myogenic potential of SMPCs (**A**) Representative immunostaining of myogenin (above) and quantitative analysis of myogenin-positive cells (below). Nuclei were counterstained with Hoechst 33258. White arrowheads indicate myogenin-positive nuclei. Scale bar = 100 μm. Proportions of myogenin-positive cells were calculated and ratios to. control values are graphed. Data are expressed as mean ± SEM (n = 4, respectively). *p < 0.05 vs. control. (**B**) Representative immunostaining of MHC (above) and quantitative analysis of MHC-positive cells (below). Nuclei were counterstained with Hoechst 33258. White arrowheads indicate MHC-positive cells. Scale bar = 100 μm. Proportions of nuclei in MHC-positive cells were calculated and ratios to control values are graphed. Data are expressed as mean ± SEM (n = 4, respectively). *p < 0.05 vs. control.

SPARC activates myotube formation [16, 17]. Thus, we examined whether decreased SPARC expression altered myogenesis in SMPCs. Young and old SMPCs were transfected with siSPARC, and myogenesis was evaluated by immunostaining for myogenin and MHC, which serve as markers for terminal myogenic differentiation. In young SMPCs, silencing of endogenous SPARC expression reduced the proportion of myogenin-positive cells (Figure 5A) and nuclei in MHC-positive cells (Figure 5B). In contrast, no statistically significant effects were observed in old SMPCs. These results suggest that SMPCs activate their myogenesis but that this stimulatory effect is lost with age.

### The responsiveness of SMPCs to exogenously added SPARC diminishes with age

**Figure 6:**
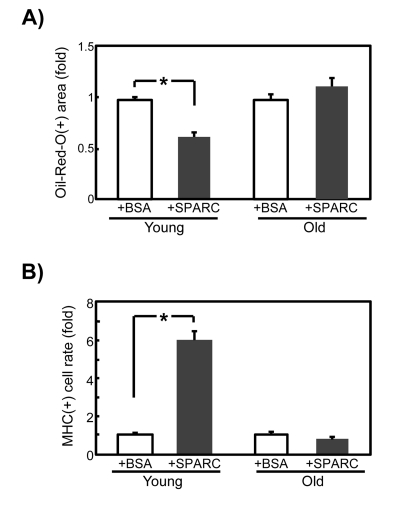
Effects of exogenously added SPARC on differentiation of SMPCs with diminished levels of SPARC expression SMPCs from young and old rats were cultured for 4 days in adipogenic media in the presence of BSA (20 μg/ml) or recombinant SPARC (20 μg/ml) 1 day after transfection with siSPARC. (**A**) Oil Red-O-positive areas were quantified as pixel values and the ratios to control values are graphed. Data are expressed as mean ± SEM (n = 3, respectively). *p < 0.05 vs. BSA-treated cells. (**B**) Proportions of MHC-positive cells were calculated and ratios to control values are graphed. Data are expressed as mean ± SEM (n = 3, respectively). *p < 0.05 vs. BSA treated cells.

We investigated whether exogenous addition of SPARC to SMPCs inhibits adipogenesis and promotes myo-genesis when endogenous SPARC is downregulated by siRNA. As shown in Figure 6, addition of 20 μg/ml SPARC decreased adipogenesis by approximately 60% (Figure 6A) and increased myogenesis by 6-fold (Figure 6B) in young SMPCs, whereas this did not affect old SMPC cultures. These results suggest that old SMPCs are less sensitive to exogenous SPARC, and further support the notion that responsiveness of SMPC to SPARC declines with age.

### Expression of the integrin alpha-5 component of the SPARC receptor is decreased on old SMPCs

**Figure 7:**
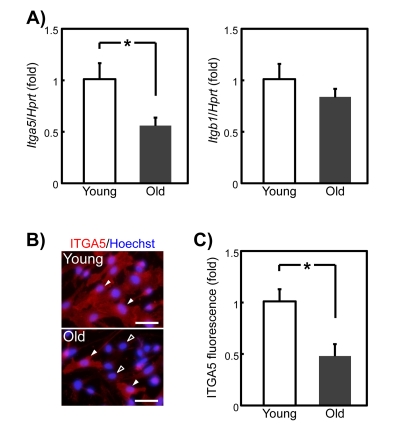
*Itga5* mRNA and protein expression in young and old rat SMPCs (**A**) qPCR analysis of *Itga5* and *Itgb1* expression in SMPCs from young and old rats 3 days after plating. Graphed data are expressed as mean ± SEM (*Itga5*: young; n = 4, old; n = 6, *Itgb1*: n = 4, respectively). *p < 0.05 vs. young. (**B**) Typical immunostaining of ITGA5 in SMPCs from young and old rats 3 days after plating. Nuclei were counterstained with Hoechst 33258. White and black arrowheads indicate intensely ITGA5-positive and slightly *ITGA5* -positive cells, respectively. Scale bar = 100 μm. (C) Quantitative analysis of relative fluorescence of ITGA5 in young and old rats SMPCs 3 days after plating. Graphed data are mean ± SEM (n = 3, respectively). *p < 0.05 vs. young rats.

Heterodimers composed of integrin α5 (ITGA5) and integrin β1 (ITGB1) serve as the receptor for SPARC on adipose stromal cells [22]. We therefore tested the possibility that the age-related decline in SPARC responsiveness is caused by a loss of ITGA5 and/or ITGB1. Quantitative PCR analysis revealed an approximately 50% decrease in the *Itga5* expression level in old compared with young SMPCs, but not *Itgb1* expression level (Figure 7A). We next determined whether decreased ITGA5 expression contributed to the age-related decline of SPARC responsiveness. Most young SMPCs were stained strongly by an ITGA5 antibody, whereas only approximately 20% of the old SMPCs were stained at an intensity similar to that of young SMPCs. The remaining cells were faintly stained (Figure 7B). We then quantified ITGA5 expression levels in young and old SMPCs by immunofluorescence. As shown in Figure 7C, fluorescence decreased by approximately 50% in old compared with young SMPCs, which is consistent with the results of qPCR.

**Figure 8:**
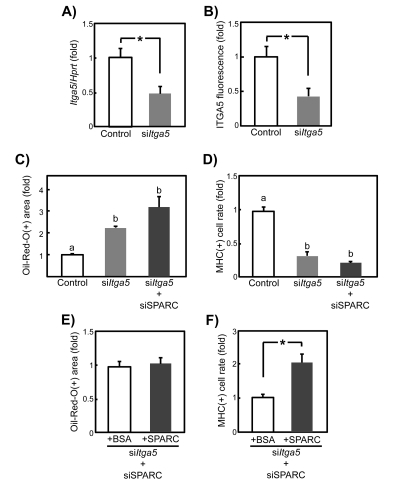
Knockdown of ITGA5 expression in young SMPCs by siRNA transfection (**A**) Efficiency of siRNA silencing was evaluated by qPCR in SMPCs from young rats 2 days after transfection with *siItga5*: Graphed data are expressed as mean ± SEM (n = 6, respectively). *p < 0.05 vs. control. (**B**) Quantitative analysis of relative fluorescence of ITGA5 in young SMPCs 2 days after transfection with si*Itga5*. Graphed data are expressed as mean ± SEM (n = 3, respectively). *p < 0.05 vs. control. Quantitative analysis of Oil Red-O staining results (**C**) and proportion of MHC-positive cells (**D**) of SMPCs at 5 days after si*Itga5* or siSPARC and si*Itga5* cotransfection. Graphed data are expressed as mean ± SEM (n = 3, respectively). Letters (a, b and c) indicate statistically significant differences (p < 0.05). Quantitative analysis of Oil Red-O staining results (**E**) and proportion of MHC-positive cells (**F**) of SMPCs cultured for 4 days in the presence of BSA (20 μg/ml) or recombinant SPARC (20 μg/ml) followed by cotransfection with siSPARC and si*Itga5*. Graphed data are expres-sed as mean ± SEM (n = 3, respectively). *p < 0.05 vs. control.

Next, we employed siRNA technology to test whether ITGA5 is involved in the role of SPARC in regulating SMPC differentiation. *Itga5* mRNA and protein expression in si*Itga5*-treated young SMPCs were evaluated by qPCR and immunocytochemistry, respectively. Transcript and protein levels decreased by approximately 50% compared with controls (Figures 8A and 8B), similar to their levels in old SMPCs (see Figure 7). *Itga5*-knockdown enhanced adipogenesis and inhibited myogenesis, and concomitant transfection of siSPARC did not significantly affect the differentiation of si*Itga5*-treated young SMPCs (Figures 8C and 8D). When ITGA5 expression was inhibited by siRNA transfection, the anti-adipogenic effect of exogenous SPARC was not observed (Figure 8E). In contrast, we detected a statistically significant 2-fold increase in myogenesis by recombinant SPARC even when ITGA5 expression was reduced (Figure 8F). Therefore, although the anti-adipogenic effect of SPARC in SMPCs almost entirely depends on ITGA5 expression, its pro-myogenic effect may also be exerted through other SPARC receptors. These findings suggest that the age-related decline in SPARC responsiveness in SMPC adipogenesis is caused by decreased ITGA5 expression, whereas other molecules are involved in decreased SPARC responsiveness during myogenesis.

## DISCUSSION

Here, we demonstrate for the first time to our knowledge that the responsiveness of SMPCs to SPARC declines with age, and may contribute to the age-related phenotypic changes in SMPCs. Because SPARC enhances myogenesis and inhibits adipogenesis [15, 16, 17], we reasoned that its decreased expression or alterations in its signaling pathway in SMPCs contribute to age-related dysfunction of skeletal muscle, such as fatty infiltration [8, 9] and impaired muscle regeneration [23]. The present study shows that the SPARC signaling pathway, rather than the level of its expression in SMPCs changes with age. It should be noted skeletal muscle cell types other than SMPCs, such as myofibers and endothelial cells, express SPARC [18]. Moreover, SPARC expression levels decline with age in the skeletal muscles of mice [24]. This indicates that although SPARC expression in SMPCs is not altered with age, the amount of SPARC available in the SMPC microenvironment would be decreased. Thus, it is possible that in addition to the decreased responsiveness of SMPC to SPARC, the age-related decline of SPARC expression levels in skeletal muscle accelerates age-related phenotypic changes in SMPC.

The present study suggests the decline in SPARC responsiveness in adipogenesis as a function of age was caused by the decreased expression of ITGA5, a subunit of the SPARC receptor of adipose stromal cells [22]. SPARC binds to ITGB1, resulting in activation of integrin-linked kinase (ILK), which interacts with the cytoplasmic tails of ITGB-subunits [25]. SPARC inhibits adipogenesis by promoting ILK activation and subsequent nuclear translocation of β-catenin [15], suggesting that the anti-adipogenic effect of SPARC is mediated by the ITGA5-ITGB1 complex. Senescent fibroblasts isolated from patients with Werner syndrome, a premature aging disorder [26], express reduced levels of ITGA5. These findings support the conclusion that ITGA5 expression levels are linked to aging, which is in agreement with our results presented here.

The present study suggests that molecules other than ITGA5 may be involved in the age-related decline in SPARC responsiveness during myogenesis. Several cell surface molecules are capable of binding to SPARC [27, 28]. Stabilin-1, a scavenger receptor, is expressed in tissue macrophages, interacts with SPARC and plays a role in the internalization of SPARC by endocytosis [27]. Vascular cell adhesion molecule (VCAM)-1, the regulator of adhesion of leukocytes to the vascular endothelium, also interacts with SPARC derived from leukocytes [28] and is expressed in C2C12 myoblasts [29]. A blocking antibody to VCAM-1 results in inhibition of myotube formation, suggesting that VCAM-1 is an element required for myogenesis of SMPCs. However, whether expression levels of either stabilin-1 or VCAM-1 in SMPCs change with age is unknown. Further studies are needed to identify the molecules that are involved in SPARC-resistance in myogenesis of SMPCs as a function of age.

It was shown that overactivation of mTOR causes feedback resistance to insulin and growth factors [30, 31]. It was suggested that signal-resistance is a hallmark of aging/senescent cells due to chronic increase in basal activity of the mTOR pathway [32, 33, 34]. In turn, such signal resistance may be abrogated by intermittent pulse-treatment with rapamycin [32]. In fact, normalization of mTOR may prevent sarcopenia [35]. Thus, the current observation that old SMPCs show refractory to SPARC may be caused by age-related overactivation of mTOR.

In summary, we show here an age-related decline in the responsiveness of SMPCs to SPARC, and suggest that this may be a cause of the age-related phenotypic changes in SMPC that could lead to IMAT accumulation. Further investigations into the nature of SPARC resistance should provide useful information for rejuvenating SMPC function in the elderly.

## METHODS

### Animals

Young (age, 4 to 5 months) and old (age, over 21 months) male Wistar-Imamichi rats were used in this study. All animals were bred in our laboratory under controlled environmental conditions: 23°C with a photoperiod of 12-h light and 12-h darkness (lights on at 0700 hours). Animals were fed ad libitum with commercial chow (Lab MR-Breeder Standard Nihon Nosan Kogyo, Yokohama, Japan). All animal experiments in this study were performed in accordance with the Guide for the Care and Use of Laboratory Animals of the University of Tokyo, and were approved by the Institutional Animal Care and Use Committee of the University of Tokyo.

### SMPC isolation and culture

SMPCs were isolated from the hindlimb and back muscles of rats according to published procedures [36, 37]. To examine adipogenic potential, SMPCs were cultured in Dulbecco's modified Eagle's medium (DMEM) containing 10% fetal bovine serum (FBS) and penicillin/streptomycin (10% FBS/DMEM) supplemented with insulin (1 μg/ml), dexamethasone (0.1 μg/ml), isobutylmethylxanthine (27.8 μg/ml), and troglitazone (10 μM, kindly donated by Daiichi Sankyo Co., Ltd.) for 2 days, followed by culture for 2 days in 10% FBS/DMEM supplemented with insulin and troglitazone, and culture for 4 days in 10% FBS/DMEM supplemented with troglitazone. To examine myogenic potential, SMPCs were cultured for 4 days in DMEM containing 2% horse serum (HS) and supplemented with antibiotics.

### Quantitative PCR (qPCR)

RNA was isolated from cells 3 days after plating using TRIzol (Invitrogen, Carlsbad, CA, USA), and cDNA was synthesized using SuperScriptII reverse transcriptase (Invitrogen). Quantitative PCR was performed using Thunderbird probe qPCR (TOYOBO, Osaka, Japan) with the probes (Roche, Basel, Switzerland) corresponding to the genes detected on Light Cycler (Roche). PCR was performed using the following primers: SPARC (forward, 5′-aggtgcagaggaaactgtcg-3′; reverse, 5′-gtttgcagtgatggttc tgg-3′), integrin α5 (*Itga5*) (forward, 5′-gcaccattcaatt tgacagc-3′; reverse, 5′-ttgtactccacaggttcctcac-3′), integrin β1 (*Itgb1*) (forward, 5′-atcatgcaggttgcagtttg-3′; reverse, 5′-cgtggaaaacaccagcagt-3′), hypoxanthine guanine phosphoribosyltransferase (*Hprt*) (forward, 5′-gaccggttctgtcatgtcg-3′; reverse, 5′-acctggttcatcatcacta atcac-3′).

### RNA interference

RNA interference was carried out with siRNA duplexes designed to target rat SPARC and rat ITGA5 (Sigma-Aldrich, St. Louis, MO, USA). Negative control siRNA (Stealth RNAi Negative Control Duplexes, Invitrogen) was also used. Two or 3 days after plating, cells were transfected with siRNAs by using Lipofectamine RNAi Max (Invitrogen) according to the manufacturer's instructions. One day after transfection, media were replaced with differentiation media in order to examine adipogenic or myogenic potential. In some experiments, recombinant mouse SPARC (120-36, PeproTech, Rocky Hill, NJ, USA) was added during adipogenic differentiation for 4 days after siRNA transfection. An equal amount of bovine serum albumin (BSA) (Sigma) was used as a control. To evaluate the efficiency of gene silencing by SPARC siRNA (siSPARC), RNA and protein samples were extracted from SMPCs 2 days after transfection, and SPARC expression was confirmed by qPCR and immunoblotting. The efficiency of gene silencing by *Itga5* siRNA (si*Itga5*) was confirmed by qPCR and immunocytochemistry 2 days after transfection.

### Immunoblotting

SMPCs were lysed in sample buffer (0.5 M Tris-HCl, 10% Glycerol, 1% sodium dodecyl sulfate (SDS), and 10% 2-mercaptoethanol). Protein extracts were separated on SDS-polyacrylamide gel and then transferred to polyvinylidene fluoride membranes. SPARC protein was detected using an anti-SPARC rabbit polyclonal antibody (A08332, Sigma-Aldrich), followed by incubation with horseradish peroxidase-labeled secondary antibody and ECL detection system (GE Healthcare Biosciences, Piscataway, NJ, USA). Blots were then reprobed with anti-β-actin antibody (mouse monoclonal antibody, A1978, Sigma) as a loading control.

### Immunocytochemistry

For immunocytochemical detection of SPARC, MyoD, myogenin, myosin heavy chain (MHC), and ITGA5, cultured SMPCs were fixed in 4% paraformaldehyde in phosphate buffered saline (PBS) at room temperature (RT) for 15 min. For detection of C/EBPα, cells were fixed in methanol. After fixation, the cells were washed 3 times with PBS, followed by blocking with 5% normal goat serum /PBS containing 0.1% Triton-X 100 (Sigma) for 20 min at RT. When methanol was used for fixation, Triton-X 100 was omitted from the blocking solution. Donkey serum was used in place of normal goat serum for the detection of SPARC. Cells were reacted with primary antibodies (described below) overnight at 4°C. After 3 washes with PBS, the cells were incubated with AlexaFluor-conjugated secondary antibodies (Invitrogen, 1:500 dilution) for 1 h at RT. Nuclei were counterstained with Hoechst 33258. Observations were performed using a fluorescence microscope (BX50, Olympus, Tokyo, Japan) equipped with a digital camera (DP70, Olympus). For quantitative analysis of differentiation, the number of SPARC-positive cells; the number of nuclei in C/EBPα-, myogenin-, or MHC-positive cells; and total nuclei were counted in 5 randomly selected fields using a 10× objective in order to calculate the proportion of positive cells. The fluorescence intensity of ITGA5 was quantified using ImageJ software (ver.1.43, NIH).

Primary antibodies and their species of origin were as follows: anti-SPARC (goat, R&D systems, Minneapolis, MN, USA, 1:100 dilution); anti-MyoD (mouse, clone A5.8, Novocastra, Newcastle upon Tyne, UK, 1:100 dilution), anti-C/EBPα (rabbit, Santa Cruz Biotechnology, Santa Cruz, CA, USA, 1:200 dilution), anti-myogenin (mouse, clone F5D, hybridoma supernatant, Developmental Studies Hybridoma Bank, *Iowa City, IA, USA*, 1:200 dilution), anti-MHC (mouse, clone MF20, hybridoma supernatant, Developmental Studies Hybridoma Bank,1:200 dilution), and anti-ITGA5 (rabbit, AB1928, Millipore, Bedford, MA, USA, 1:1000 dilution).

### Oil Red-O staining

Oil Red-O staining was used for detection of accumulated cellular lipid droplets, which represent the adipogenic potential of SMPCs. SMPCs were fixed in 4% paraformaldehyde in PBS for 15 min at RT, rinsed 3 times with PBS, stained with Oil Red-O mixture (2:3 mixture of 0.5% (w/v) Oil Red-O (Sigma) in 2-propanol and distilled water) for 10 min. For quantification of adipogenesis, 5 different fields randomly chosen under the microscope using a 10× objective were photographed, and the areas stained with Oil Red-O were quantified using ImageJ software. Values representing the incidence of adipogenesis were expressed as mean pixel measurements.

### Statistical analyses

ANOVA followed by Tukey-Kramerpost-hoc comparisons were used to evaluate statistical differencesamong the groups. Student's *t*-test was used to examine differences between the 2 groups. P values less than 0.05 were considered statistically significant.

## Abbreviations

SMPC: skeletal muscle progenitor cells
IMAT: intramuscular adipose tissue
SPARC: Secreted protein acidic and rich in cysteine
